# Primary Chest Wall Angiofibroma: Expanding the Spectrum of Benign Chest Wall Tumours

**DOI:** 10.7759/cureus.96199

**Published:** 2025-11-06

**Authors:** Supraja Ramesh, Hasanali D Walji, Andrew Robinson, Antonio E Martin-Ucar

**Affiliations:** 1 Cardiothoracic Surgery, University Hospitals Coventry and Warwickshire NHS Trust, Coventry, GBR; 2 Pathology, University Hospitals Coventry and Warwickshire NHS Trust, Coventry, GBR

**Keywords:** angiofibromas, benign tumours, cardio thoracic surgery, chest wall tumours, vascular neoplasm

## Abstract

Angiofibromas are benign vascular tumours predominantly found in the nasopharynx, with primary chest wall occurrences being exceedingly uncommon. While well-documented in certain anatomical locations like the nasopharynx and facial skin, primary chest wall angiofibromas represent an extremely rare entity with limited information in the medical literature. Management should follow principles for benign but highly vascular chest wall tumours, with comprehensive imaging, cautious biopsy, and complete surgical excision when feasible. Recognising this rarity expands the differential diagnosis of chest wall masses and underscores the importance of documenting atypical sites to improve future diagnosis and management. We report the case of a 62-year-old woman with a symptomatic primary chest wall angiofibroma treated through surgical resection. The patient made an excellent recovery following surgery, which reinforces the clinical importance of identifying rare benign vascular tumours early to guide timely and effective management.

## Introduction

Angiofibromas are benign, highly vascular tumours marked by a rich vascular network embedded in fibrous tissue [1]. They are most frequently encountered in the nasopharynx of adolescent males and less commonly in association with tuberous sclerosis complex as facial papules [2-5]. Occurrence of angiofibromas beyond these sites is extremely rare, with chest wall involvement scarcely reported to date in medical literature, which makes each instance valuable for expanding clinical knowledge and guiding management. Chest wall masses encompass a wide differential, including benign or malignant lesions; however, angiofibroma is rarely considered [6]. This report explores the case of a 62-year-old woman who developed a primary chest wall angiofibroma, necessitating surgical intervention. 

## Case presentation

A 62-year-old woman presented with a progressively enlarging, painful swelling over the left lower lateral chest wall that began four years prior to admission. The mass had steadily increased in size and was associated with worsening dyspnoea and nocturnal pain despite analgesia. On examination, a firm, tender lesion fixed to the underlying structures was palpable without overlying erythema or discharge.

**Figure 1:**
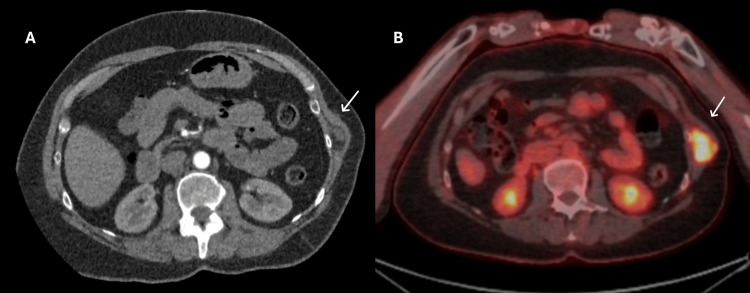
A: CT of the thorax and B: PET-CT A: CT of the thorax showing a well-defined, hypervascular mass on the left chest wall arising from the intercostal space of the 10th rib (arrow). B: PET-CT demonstrating a 3-cm left chest wall lesion with increased metabolic activity (SUVmax 13.1), consistent with a hypervascular soft tissue tumour (arrow).

Initial ultrasound demonstrated a well-defined, heterogeneously echo textured mass. CT of the thorax confirmed a hypervascular mass measuring 2.8 x 2.8 x 3.4 cm (feeding vessel: intercoastal artery of the 10th rib) (Figure 1A). PET imaging revealed a 3 cm lesion with a maximum standardised uptake value (SUVmax) of 13.1 (Figure 1B). Given the progressive symptoms and radiological features, the patient underwent complete surgical excision of the tumour along with segmental resection of the 10th rib.

**Figure 2:**
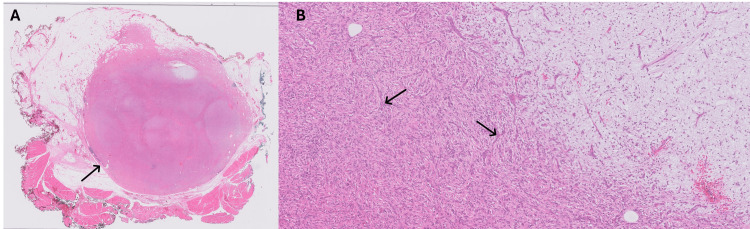
A: Low-power photomicrograph (0.5×). B: High-power photomicrograph (10×) A: Low-power photomicrograph (0.5×) showing a well-circumscribed angiofibroma (arrow) within the chest wall with adjacent skeletal muscle. The tumour shows infiltration of adipose tissue. B: High-power photomicrograph (10×) showing spindled cells (arrows) within a rich, delicate, vascular network. Foci of the tumour show myxoid stromal change on the right side.

Histopathology demonstrated a well-circumscribed angiofibroma of soft tissue within the chest wall adjacent to skeletal muscle with infiltration of adipose tissue (Figure 2A). High-power microscopy showed spindled cells in a delicate vascular network with focal myxoid change (Figure 2B). Next-generation sequencing detected an in-frame AHRR: NCOA2 gene fusion between exon 9 of AHRR and exon 16 of NCOA2, which is a recurrent finding in angiofibroma of soft tissue.

The patient recovered uneventfully, and was discharged on her postoperative day 2, with resolution of her pain and dyspnoea. Currently, she is being followed up in clinic with no evidence of recurrence till date.

## Discussion

Angiofibromas are benign fibrovascular tumours composed of a rich network of thin-walled blood vessels embedded in a collagenous stroma containing stellate and spindle-shaped fibroblasts [1]. Most are encountered as juvenile nasopharyngeal angiofibromas (JNAs), primarily in adolescent males, arising from the sphenopalatine foramen and potentially extending into surrounding structures [2,3]. Angiofibromas also occur as facial papules in patients with tuberous sclerosis complex (TSC), where they represent a major diagnostic criterion and may occasionally extend to the neck or upper trunk [4,5]. However, these tumours are extensions of the facial distribution rather than true primary chest wall lesions.

Soft tissue tumours of the chest wall encompass a broad histological spectrum, including malignant fibrous histiocytoma, liposarcoma and fibrosarcoma [6]. Angiofibroma, by contrast, is not listed among common entities in large series or reference texts, and a thorough review of the literature reveals a notable absence of well-documented case reports or series specifically describing primary chest wall angiofibromas, unlike JNAs or facial angiofibromas in TSC, which have extensive literature support. This absence of reports suggests that either primary chest wall angiofibromas are extremely rare occurrences, misclassified under other soft tissue tumours/ fibrovascular neoplasms, or occur as secondary extensions from other sites rather than as true primaries.

Comparative review of the limited literature shows that most previously reported angiofibromas outside the nasopharynx arise in the extremities or pelvic soft tissue, often in middle-aged adults, and share similar histologic and molecular features, particularly the AHRR-NCOA2 gene fusion, thereby excluding other spindle cell neoplasms [1,2]. However, unlike those lesions, our case represents a rare intrathoracic manifestation confined to the chest wall without extension into adjacent structures. Compared with other benign chest wall tumours such as desmoid-type fibromatosis and neurofibroma, angiofibroma displays a more indolent clinical course and lacks the infiltrative or neural differentiation typically seen in those entities [7]. This distinction is clinically relevant, as it influences the extent of surgical excision and postoperative follow-up strategy.

Radiological features of angiofibromas in other sites typically include intense contrast enhancement on CT and MRI because of their hypervascularity [3]. In the chest wall, cross-sectional imaging with CT or MRI is essential to delineate the extent and potential involvement of underlying structures. Biopsy remains the gold standard for definitive diagnosis, although it should be approached with caution in highly vascular lesions. In our patient, PET-CT confirmed hypermetabolic activity and vascular supply from the intercostal artery, prompting definitive resection.

In the absence of specific guidelines on chest wall angiofibromas, treatment recommendations must be extrapolated from approaches to angiofibromas in other locations and general management principles for benign chest wall tumours. For JNAs, surgical excision remains the mainstay of treatment, often preceded by preoperative embolisation to reduce intraoperative bleeding [3,8,9]. For soft tissue tumours of the chest wall, wide surgical excision with an emphasis on achieving negative margins is generally recommended [6]. Our patient underwent complete resection with segmental rib excision and experienced prompt symptom relief without complications.

This case underscores the diagnostic challenges posed by such rare presentations with limited literature and the importance of considering unusual differential diagnoses like angiofibroma when evaluating chest wall masses. Documentation of such cases is crucial to expanding clinical awareness and contributing to the growing knowledge of rare benign fibrovascular tumours in atypical locations.

## Conclusions

Primary chest wall angiofibroma is an exceptionally rare benign tumour, representing an entity seldom encountered in clinical practice. This case emphasises the need to include vascular fibroblastic lesions in the differential diagnosis of chest wall masses. Accurate preoperative characterisation through imaging and cautious biopsy can aid in avoiding misdiagnosis or overtreatment. Complete surgical excision remains the cornerstone of management and is associated with excellent outcomes. Reporting such rare entities enhances diagnostic accuracy and supports evidence-based management of atypical chest wall tumours.
